# The effect of wet cupping on reactive airway dysfunction syndrome (RADS) caused by early exposure to chlorine gas: A randomized clinical trial

**DOI:** 10.22038/AJP.2024.25107

**Published:** 2025

**Authors:** Khatereh Akbari Mashak, Khosro Agin, Elham Emaratkar, Mohammad Gholami Fesharaki, Hasan Namdar

**Affiliations:** 1 *Department of Traditional Persian Medicine, School of Persian Medicine, Shahed University, Tehran, Iran *; 2 *Department of the Pulmonary Medicine, Loghman Hakim Hospital, Faculty of Medical, Shahid Beheshti University, Tehran, Iran*; 3 *Department of Biostatistics, Faculty of Medical Sciences, Tarbiat Modares University, Tehran, Iran*; 4 *Traditional Persian Medicine Clinical Trial Research Center, Shahed University, Tehran, Iran*

**Keywords:** Reactive airway dysfunction syndrome, Persian Medicine, Bleaching agents, Chlorine gas, Hijamat, Wet cupping

## Abstract

**Objective:**

In the absence of targeted antidotes for chlorine gas poisoning, a common yet concerning problem, this study investigates the effect of Wet Cupping Therapy (WCT, or “*Hijamat”*) on the recovery process in chlorine-induced reactive airway dysfunction syndrome (RADS) patients.

**Materials and Methods:**

This randomized controlled trial enrolled 24 patients experiencing acute inhalation of chlorine poisoning in Tehran, Iran (2020-2021). Patients were randomly divided into control (n=12, receiving conventional treatment) and intervention (n=12, receiving conventional treatment plus WCT) groups. Signs and symptoms were assessed pre-intervention, and in the first hour, first week, and first month post-intervention.

**Results:**

Medical records of 24 patients, including 3(12.5%) men and 21(87.5%) women, with a mean age of 42.92 years old, were evaluated. Baseline characteristics were similar between the groups. WCT significantly improved symptoms (dyspnea, cough, chest tightness, etc.) within the first hour (p=0.003) compared to the controls, with no future significant changes during the first week and first-month post-WCT. Comparison between the groups revealed substantial differences in the following variables: dyspnea scale (p=0.009), respiratory rate (p=0.026), cough (p=0.001), breath shortness (p=0.006), chest tightness (p=0.002), chest pain (p=0.010), substernal burning (p=0.015), throat sore (p=0.005) and hoarseness (p=0.027). Peak flow meter readings, reflecting lung function, were also significantly higher in the WCT group at all time-points (p<0.007).

**Conclusion:**

WCT may offer a rapid and sustained improvement in pulmonary and respiratory symptoms following acute chlorine inhalation injury.

## Introduction

 Reactive airway dysfunction syndrome (RADS), an asthma-like stimulating syndrome typically manifesting within 24 hours of accidental exposure to a chemical respiratory irritant (Brooks et al., 1985), is a known complication of chlorine gas inhalation. Chlorine gas, a widely used toxicant in industrial, water treatment, and even domestic settings is a well-established cause of RADS (Zhou et al., 2018). In the United States alone, chlorine gas exposure prompts approximately 9,000 calls to poison control centers annually (Carlisle et al., 2016). Notably, most household chlorine poisonings occur when chlorine-containing bleach, such as sodium hypochlorite, accidentally mixes with acidic detergents (including those containing hydrochloric acid or ammonia) in confined spaces like bathrooms or toilets (Gorguner et al., 2004).

Despite the increasing incidence of chlorine gas poisonings, obtaining accurate prevalence data remains challenging due to limitations in outpatient referral capture and the absence of mandatory reporting by medical centers. Scattered reports from various sources, including articles and news agencies, highlight instances of group poisonings arising from accidental exposures, particularly in pool environments (Beigoli et al., 2024; Masoumi et al., 2022). Inhalation of chlorine gas at concentrations exceeding 1ppm can irritate the upper airways (Rotman et al., 1983), potentially leading to a cascade of complications including hypoxemia, upper and lower airway obstruction, dyspnea, acute pulmonary edema, pneumonitis, and even acute respiratory distress syndrome (ARDS) )Samal et al., 2010(. However, conventional medicine currently lacks a specific antidote for chlorine gas poisoning (Zhou et al., 2018). Standard conventional treatment primarily focuses on supportive measures (Zellner and Eyer, 2020), including oxygen administration, bronchospasm management with beta-agonists (White and Martin, 2010), and insevere cases, mechanical ventilation via intubation (Evans, 2005; Winder, 2001).

While various pharmaceutical therapies, including corticosteroids (Carlisle et al., 2016), nitrite therapy (Honavar et al., 2017), ascorbate, N-acetylcysteine (Yadav et al., 2010), antioxidants (Olman et al., 2004), nitric oxide regulators (Honavar et al., 2011), and high molecular weight hyaluronan (Zhou et al., 2018) have shown promise in animal models, their efficacy in humans remains unproven. These treatmentsare not currently approved for clinical use (Summerhill et al., 2017). Therefore, primary management of chlorine-induced RADS relies on implementing lung-protective ventilation techniques, characterized by low tidal volume, to minimize the risk of hypoxemiaand hypercapniawhich can exacerbate ARDS development (Huppert et al., 2019; Villar et al., 2006; Ware and Matthay, 2000).

Chlorine gas's high water solubility and rapid conversion to hydrochloric acid (Man et al., 2017) upon contact with extensive lung tissue facilitate its diffusion through the porous endothelium of the alveolar-blood barrier and into the bloodstream, a process that is further aggravated by compromised epithelial and endothelial barrier integrity (Huppert et al., 2019). These factors necessitate immediate interventions to detoxify the blood and minimize potential long-term sequelae from the initial insult and subsequent organ damage.

The historical burden of human exposure to various poisons has driven the development of diverse therapeutic approaches across different medical traditions (Rezaei et al., 2023; Mahdizadeh et al., 2015). Persian Medicine (PM), a longstanding and holistic system of Iranian traditional medicine, offers a unique perspective on managing poisonings (Mousavi et al., 2020; Nasiri et al., 2023a, 2023b). With a rich history spanning millennia, PM boasts significant contributions from scholars such as *Muhammad ibn Zachariah al-Razi*, *IbnSina*, and *EsmaeilJorjani* (Aghabeiglooei et al., 2023).

Wet Cupping Therapy (WCT), also known as “*Hijamat”*, is a traditional detoxification method within PM with potential applications in managing chlorine gas poisoning (Azam-Khan, 1869). The procedure involves controlled suction and scarification of the skin at specific body areas, followed by bloodletting (El-Sayed et al., 2013). In PM, WCT applied between the shoulders (*Hijamat-e-kahal*), has been advocated for respiratory ailments like cough and bloody sputum (Avicenna, 1998). While the exact mechanism of action for WCT remains unclear, several studies suggest promising effects (Cao et al., 2012; Eghbalian et al., 2019; Kenari et al., 2022; Kordafshari et al., 2017). Especially, lung diseases improvement and enhancing respiratory function havebeen substantiated by numerous studies (Hong et al., 2006; Zhao et al., 2012). WCT has been shown to increase arterial blood oxygen saturation (SaO2) in other patient populations (Aleyeidi et al., 2015; Ali-Ismail et al., 2021; Hekmatpou et al., 2013; Seydi et al., 2023), potentially aiding in hypoxia caused by chlorine gas exposure. Additionally, WCT's reported ability to reduce oxidative stress (Akbari et al., 2013) and modulate the immune system (Baghdadi et al., 2015; Khalil et al., 2013; Molavi et al., 2017; Soleimani et al., 2020; Widada, 2017) aligns with the physiopathology of chlorine gas injurywhich involves inflammatory processes and oxidative damage (Baghdadi et al., 2015; Tagil, et al., 2014). Furthermore, evidence suggests potential detoxification effects of WCT including the elimination of heavy metals from the body (Umar et al., 2018), removal of blood impurities (Tahmasebi et al., 2015) and treatment of aluminum phosphide poisoning (Fallah, 2016).

The advantageous anatomical location of“*Hijamat-e-kahal*”, encompassing brown fat tissue (Yao et al., 2011), and proximity to sympathetic ganglia, the thoracic duct, and major vessel divisions, suggests potential benefits for the heart, lungs, chest, and neck lymphatic system with minimal complications (Ghods et al., 2016). Furthermore, the convergence of five meridians in this region according to Traditional Chinese Medicine (TCM) theory (Wang and Wang, 2008) might promote improved energy flow following WCT (Kim et al., 2011). 

The physiopathology of RADS, characterized by acute pulmonary edema, is understood in PM as“*varam-e-harr-e-riyavi*” (i.e. hot pulmonary edema). Traditional treatments for this condition include bloodletting, WCT, or leech therapy, followed by the administration of "cold temperament" foods and medications to counteract the heat (Azam-Khan, 1869). A previous review article (Akbari-Mashak et al., 2023) explored the connection between RADS and PM's approach to hot pulmonary edema. Building on this concept, we observe the parallels between the inflammatory nature of RADS (considered "hot" by Avicenna in PM theory) and the theorized anti-inflammatory or "cooling" effects of WCT. To investigate this potential link, we designed a clinical trial to examine the efficacy of “*Hijamat-e-kahal”* in improving the condition of patients with RADS caused by chlorine gas exposure.

## Materials and Methods

### Study design

This study employed a randomized, controlled clinical trial (RCT) design. Twenty-four patients diagnosed with RADS following acute chlorine gas exposure from domestic bleach misuse were recruited from Loghman-e-Hakim Hospital in Tehran, Iran, during 2020- 2021. The diagnosis of chlorine poisoning was established based on the patient's self-reported exposure history, clinical manifestations, and findings from clinical examinations. Blinding was not possible due to the nature of the intervention.

### Ethics statement

The research procedure adhered entirely to the guidelines set forth by the Human Ethics Committee of Shahed University (Date: 2020-02-17) and was implemented with an ethical code: IR.SHAHED.REC.1398.130. Additionally, this trial was approved by the Iranian Registry of Clinical Trials. Code of IRCT: 20200309046727N1. 

### Participants, randomization, and sampling

**Figure 1:**
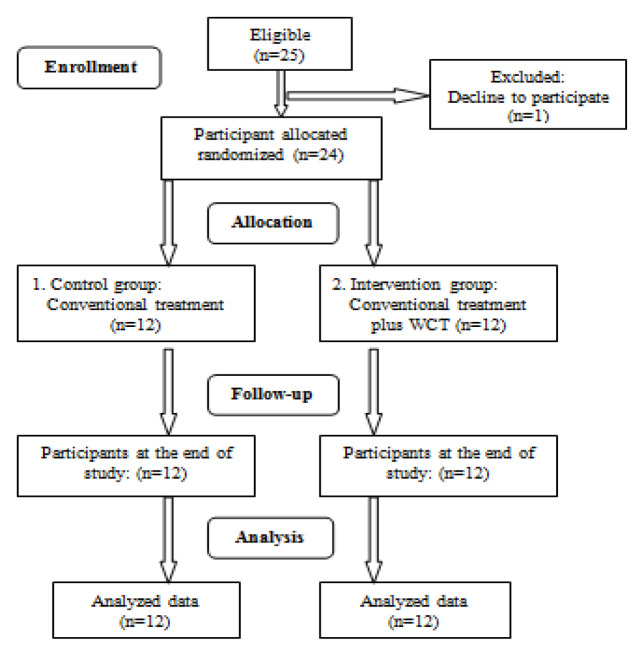
The CONSORT (consolidated standards of reporting trials) flow diagram of the study. This diagram outlines the flow of participants throughout the various stages of the trial, including enrollment, allocation to intervention or control groups, follow-up, and analysis. *WCT: Wet cupping therapy.

The sample size of 24 participants was calculated considering a first-type error of 0.05 and an effect size based on the findings of our pilot study. Following signing the informed consent forms and simple randomization (computer-generated random number table), 12 patients were allocated to each of the two groups as follows: 

Group 1 received conventional treatment alone (control group), while Group 2 received conventional treatment plus WCT (intervention group). Conventional treatment adhered to the clinical guidelines outlined by the Ministry of Health of Iran (2017) for managing chlorine gas poisoning (Table 1). In the interventional group, WCT was administered following conventional treatment. The study flowchart is illustrated in Figure 1 .

The inclusion criteria were age of 20 to 60 years old; confirmed chlorine gas exposure within 24 hours, as per Brookes' criteria (Shakeri et al., 2008); absence of pre-existing chronic medical conditions such as diabetes, heart failure, renal failure, asthma, chronic obstructive pulmonary disease (COPD), or mental disorders; not being pregnant, lactating, or menstruating; no history of hypersensitivity disorders including urticaria, atopy, or eczema; not taking anticoagulant medications like aspirin, warfarin, clopidogrel, and rivaroxaban (Xalerto).

The exclusion criteria were refusal to participate in the study; RADS diagnosis due to chemical irritants besides chlorine gas; presence of contraindications for standard blood sampling procedures; contraindications for WCT within the past 12 hours according to PM, including females undergoing menstruation, participants experiencing heavy bleeding or individuals with severe weakness; co-morbidities of potential confounding conditions, particularly with pulmonary effect or inflammatory response (e.g. influenza, COVID-19, pneumonia, etc.), during the one-month study period.

### Intervention

WCT was performed using disposable plastic cups with a 120 ml volume and manual suction. Following a standardized protocol, participants were seated in a comfortable position, and the targeted area between the shoulder blades on the upper back, specifically at the level from T2 to T4 vertebrae, was suctioned with the cup for 3-5 min. Subsequently, superficial incisions were approximately 5-7 mm long and with a depth of 1-2 mm on the skin surface- using a sterile number 15 scalpel blade. The cup was replaced, and the site was suctioned again for another 3-5 min. This process was repeated for a total of three suction cycles. Approximately 15-30 ml of blood was collected during this procedure.

### Measurement

Data were collected using pre-designed forms developed by an expert panel, incorporating relevant sections from previous studies (Brooks et al., 1985; Gorguner et al., 2004). The forms collected demographic characteristics, medical history, co-morbid conditions, presenting signs and symptoms, and examination findings. Data meeting the inclusion and exclusion criteria were included in the final analysis.

In both groups, dyspnea scores were assessed using the validated Medical Research Council (MRC) scale score (Williams, 2017). 

We also measured respiratory rate (RR), pulse rate (PR), systolic blood pressure (SBP), diastolic blood pressure (DBP), and oxygen saturation (SpO2) using a finger pulse oximeter (Beurer-P030 model), along with peak flow meter (Rossmaxmodel: PF120A, 60-800 L/min, Swiss GmbH, Tramstr. 16, CH-9442 Berneck, Switzerland) readings. These assessments were conducted at baseline, then at intervals during the first hour, and again at one week and one-month post-intervention.

The severity of patient-reported symptoms including shortness of breath, cough, chest tightness, sore throat, chest pain, hoarseness, substernal burning, nausea, vomiting, dizziness, confusion, headache, burning nose, rhinorrhea, eye irritation, blurred vision, skin irritation, and bloody sputum, was qualitatively recorded at baseline and three subsequent time-points following the intervention in both groups. Changes in symptom severity, symptom duration, and recovery time of signs, symptoms, and clinical assessments were considered secondary outcomes.

### Statistical analysis

Statistical analyses were performed using R statistical software version 3.1.2 (R Project for Statistical Computing, Vienna, Austria), with a significance level set at p<0.05. The patients symptoms were scored on a scale of 1 (no symptom) to 5 (very severe symptom). The total symptom scores of each questionnaire divided by the number of symptoms were calculated as the mean symptom scores, compared at all time-points in the two groups.

Additionally, the MRC dyspnea scales were scored on a scale of 1(without shortness of breath except on strenuous exercise) to 5 (severe breathlessness during an usual activity). Descriptive statistics were used to summarize the data. Continuous variables were assessed for normality using the Shapiro-Wilk test. Qualitative variables are reported as frequency (percentage), while normally distributed data are expressed as mean ± standard deviation (±SD). Differences between the groups were assessed using the Mann–Whitney U or T test for continuous variables and the Chi-square test or Fisher’s exact test for categorical variables. For variables measured repeatedly over time (e.g. SpO2), the Generalized Estimating Equation (GEE) regression was employed. A p-value of ≤0.05 was considered statistically significant in this study.

## Results

### Baseline characteristics of patients

Twenty-one women and three men participated in this study with a mean age of 42.92 years (range=26 to 60 years, SD=9.6). The two groups were matched to age (control group: 44.25±9.36, intervention group: 41.58±10.08 years, p=0.509) and sex (control group: 11 [91.7%] women vs. intervention group: 10 [83.3%] women, p=0.537). The locations of chlorine exposure were primarily enclosed toilets and bathrooms (n=15), followed by open areas (n=6), workplaces (n=2), and one incident that occurred in a sauna pool (n=1).

**Figure 2:**
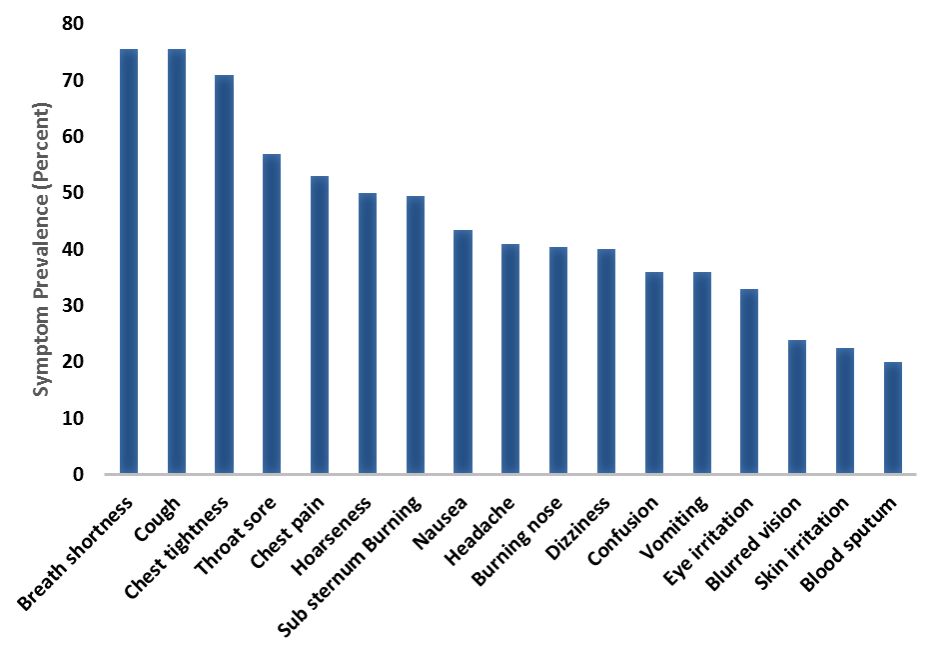
Prevalence of symptoms reported among all patients poisoned with chlorine gas pre-intervention. Data were presented with frequencies and percentages (%). Complete baseline symptoms data were available for 24 patients who participated in the study.

Among all patients, the most prevalent pre-intervention symptoms reported were shortness of breath (75.5%), cough (75.5%), chest tightness (71%), sore throat (57%), chest pain (52.5%), hoarseness (50%), sub-sternum burning (49.5%), nausea (43.5%), headache (41%), burning nose (40.5%), dizziness (40%), vomiting (36%), confusion (36%), eyes irritation (33%), blurred vision (24%), skin irritation (22.5%), and bloody sputum (20%) (Figure 2).

### Primary outcome

**Figure 3:**
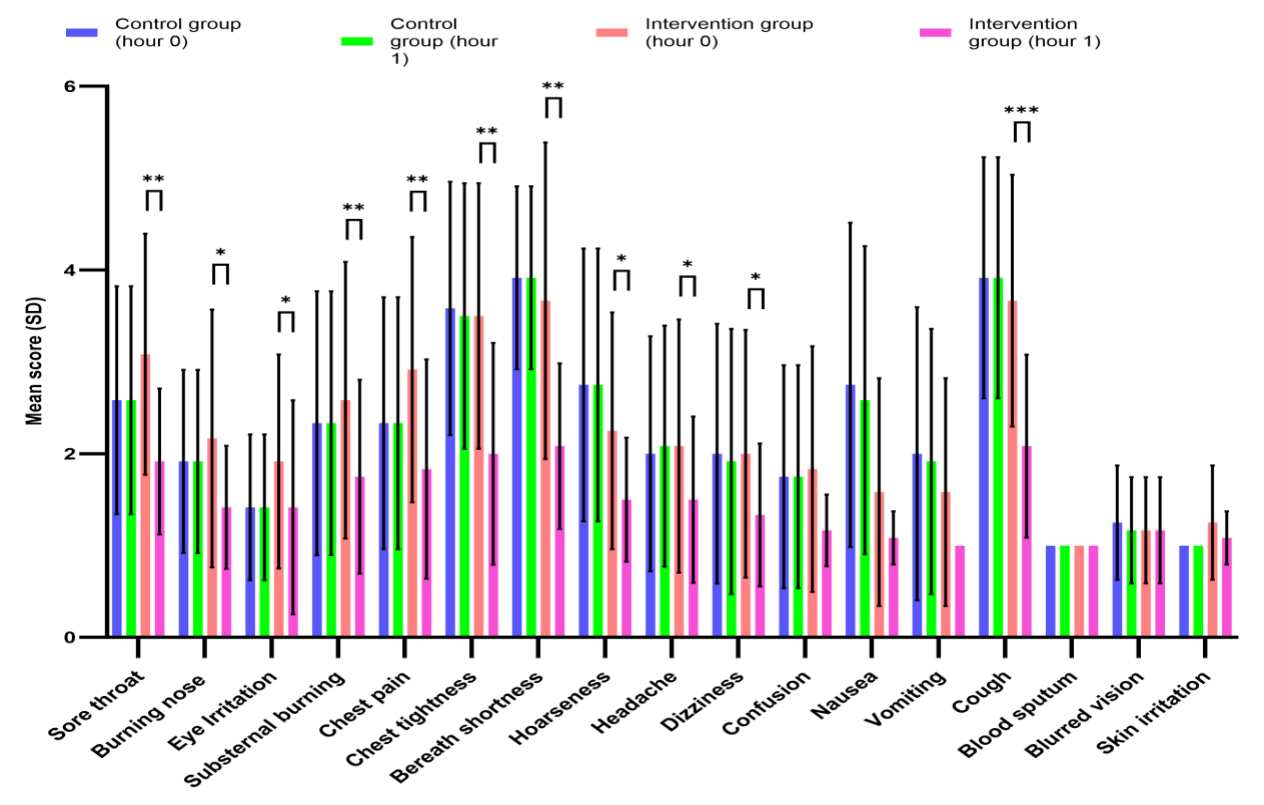
Comparison of mean symptom scores betweentwo groups poisoned with chlorine gas in the first hour post-intervention. Data are reported as mean ± (SD), SD: Standard deviation. The within-group p-values for changes in symptoms of each group (12 patients in each group) in the first hour after the intervention were analyzed using a paired sample t-test. In the intervention group, the mean score decreased significantly, and the p-value showed significant changes, including: sore throat (p=0.002), burning nose (p=0.032), eye irritation (p=0.026), sub sternum burning (p=0.005), chest pain (p=0.002), chest tightness (p=0.001), shortness of breath (p=0.001), hoarseness (p=0.012), headache (p=0.027), dizziness (p=0.013), and cough (p<0.001). However, in the control group, none of the mean scores for symptoms decreased significantly. * indicates p<0.05, ** indicates p<0.01, and *** indicates p<0.001.

One hour after the intervention, the mean score for all symptoms significantly decreased in the intervention group from 2.25 to 1.49 for before and first hour post-intervention, respectively, (95% CI of difference=0.49-1.02, p<0.001). Conversely, the mean symptom score in the control group did not change significantly during the same period (2.26 vs. 2.24 for before and first hour post-intervention, respectively, p=0.096). Further analysis of individual symptoms revealed significant improvements in the WCT group for sore throat (3.08 vs. 1.93, p=0.002), burning nose (2.17 vs. 1.42, p=0.032), eye irritation (1.92 vs. 1.42, p=0.026), sub-sternum burning (2.58 vs. 1.75, p=0.005), chest pain (2.92 vs. 1.83, p=0.002), chest tightness (3.5 vs. 2.0, p=0.001), shortness of breath (3.67 vs. 2.08, p=0.001), hoarseness (2.25 vs. 1.5, p=0.012), headache (2.08 vs. 1.50, p=0.027), dizziness (2.0 vs. 1.33, p=0.013), and cough (3.67 vs. 2.08, p<0.001) (for intervention and control, respectively, all p-values<0.05). In contrast, the control group did not show any significant decreases in symptom scores (Figure 3).

**Figure 4:**
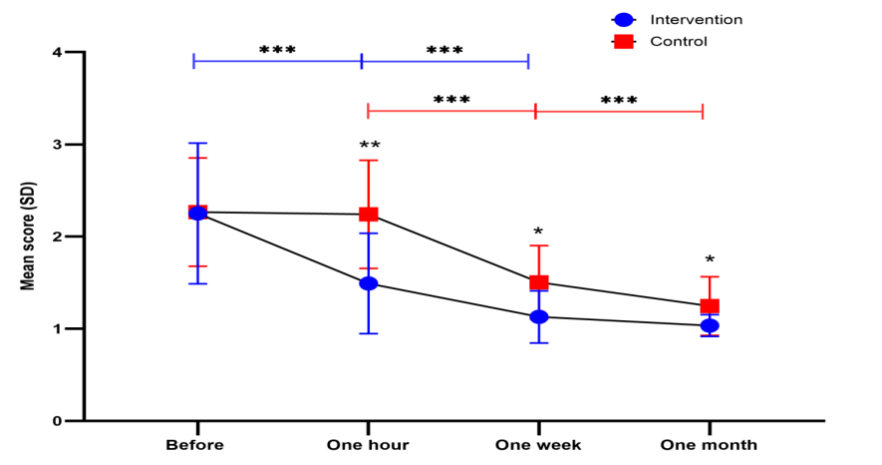
Trend of mean total symptom scores during follow-up intwo groups of patients poisoned with chlorine gas. Data are reported as mean ± (SD), SD: Standard deviation. The mean symptom scores were calculated by the adding the total symptom scores of each questionnaire divided by the number of symptoms. Dots on the graph represent the mean score, and the error bars depict the standard deviation. The difference between the two groups, each containing 12 patients, was analyzed using an independent samples t-test. The score was lower in the intervention group compared to with the control group at all time-points: first hour (1.49 vs. 2.24 for intervention and control, respectively, p=0.004), first week (1.12 vs. 1.50 for intervention and control, respectively, p=0.014), and first month (1.03 vs. 1.24 for intervention and control, respectively , p=0.043) after intervention, while the score wasnot significantly different between the groups before the intervention (2.24 vs. 2.26 for intervention and control, respectively, p=0.958).* indicates p<0.05, ** indicates p<0.01, and *** indicates p<0.001. The blue line indicates inter-group significance in intervention and the red line indicates inter-group significance in control.

Following the initial improvement observed one hour after the intervention, the mean symptom score continued to decrease in both groups between one hour and one- week post-intervention (intervention group: 1.49 vs. 1.12 for one hour and one-week post-intervention, respectively, 95% CI of difference =0.18-0.54, p=0.001; control groups: 2.24 vs. 1.50 for one hour and one-week post-intervention, respectively, 95% CI of difference =0.48-0.99, p<0.001). Interestingly, the control group exhibited further improvement in symptom scores between one week and one-month post-intervention (1.50 vs. 1.24 for one week and one-month post-intervention, respectively, 95% CI of difference =0.13-0.38, p=0.001). In contrast, the intervention group's scores remained stable during this period (1.12 vs. 1.03 for one week and one-month post-intervention, respectively, p=0.086) (Figure 4). This finding suggests that WCT may lead to a more rapid improvement in symptoms, with the control group eventually catching up over time.

Notably, the mean score for all symptoms was significantly lower in the intervention group compared to the control group at all measured time points, including the first hour (1.49 vs. 2.24 for intervention and control, respectively, p=0.004), first week (1.12 vs. 1.50 for intervention and control, respectively, p=0.014), and first month (1.03 vs. 1.24 for intervention and control, respectively, p=0.043) after the intervention. However, the scores were equal between the groups before the intervention (2.24 vs. 2.26 for intervention and control, respectively, p=0.958) (Figure 4).

### Secondary outcome

Detailed information about all study variables in both groups at each time-point is presented in Table 2. As expected, none of the variables exhibited significant differences between the intervention and control groups before the intervention. 

The MRC Dyspnea Scale was significantly lower in the intervention group than the control group (2.0 vs. 3.58, for intervention and control, respectively, p=0.02). This finding suggests that the intervention group experienced less breathlessness than the control group post-WCT. The difference in dyspnea scores remained statistically significant in the follow-up stages (1.25 vs. 1.92 for intervention and control, respectively, in the first week and 1.08 vs. 1.50 for intervention and control, respectively, in the first-month follow-ups, p<0.05), indicating a sustained improvement in the intervention group.

Peak flow meter readings were also significantly higher in the intervention group compared to the control group at all measured time-points post-intervention (first hour: 263.3 vs. 165.8 for intervention and control, respectively, p=0.003; first week: 291.6 vs. 212.55 for intervention and control, respectively, p=0.008; first month: 325.0 vs. 233.3 for intervention and control, respectively, p=0.002). These findings suggest that WCT may have improved lung function in the intervention group compared to the control group.

While SpO2 levels were not comparable between the intervention and control groups one hour after the intervention (96.83% vs. 96.58% for intervention and control, respectively, p=0.798), SpO2 was significantly higher in the intervention group compared to the control group at both the first week (97.42% vs. 95.33%for intervention and control, respectively, p=0.023) and first month (98.0% vs. 95.33% for intervention and control, respectively, p=0.001) follow-up assessments. This finding suggests that WCT may have improved oxygenation over time in the intervention group.

In contrast, the respiratory rate were significantly lower in the intervention group than the control group one hour after the intervention (20.0 vs. 34.67 for intervention and control, respectively, p=0.026), and remained no difference in the follow-up stages (16.0 vs. 16.33 for intervention and control, respectively, p=0.065 in the first week and 14.0 vs. 15.33 for intervention and control, respectively, p=0.093 in the first-month). This finding suggests that WCT may have enhanced lung ventilation faster in the intervention group than in the control group.

Additionally, no significant differences were observed in other variables, including pulse rate and blood pressure, during the first month.

### Adverse effects

 All participants were monitored for adverse effects during and following the intervention. No serious complications were reported. A few participants experienced mild, transient discomfort at the application site, such as localized redness or a burning sensation. These symptoms resolved spontaneously within three days.

## Discussion

This study confirms previous findings on the primary symptoms of chlorine-induced RADS, including dyspnea, cough, wheezing, shortness of breath, chest tightness, chest pain, headache, dizziness, sore throat, irritation of the eyes and nose, sputum (Boskabady et al., 2014; Ejaz et al., 2022; Gorguner et al., 2004). All patients reported experiencing these symptoms within minutes or hours of chlorine exposure (Figure 1).

Furthermore, WCT significantly reduced these symptoms compared to the control group, particularly within the first hour. This improvement was further corroborated by positive changes observed in MRC Dyspnea Scale scores, Peak Flow Meter readings, and oxygen saturation. These findings align with existing research demonstrating the efficacy of both dry and wet cupping for cough and asthma (Joushan et al., 2020). Additionally, two previous studies suggest a potential benefit of cupping therapy in enhancing immunological function in patients with stable chronic obstructive pulmonary disease (COPD) (Xiao et al., 2010; Zhang et al., 2006). 

This finding aligns with the theoretical framework proposed by Al-Bedah et al. (2019), which suggests that WCT has the potential to reduce pain, exert anti-inflammatory effects, and enhance blood circulation, alongside immune modulation and hematological adjustments.

Ali-Ismail et al. (2021) reported that cupping therapy has immediate effects on various physiological parameters including blood pressure, oxygen saturation, pulse rate, and chest expansion. However, our study design precluded the evaluation of blood pressure changes due to the absence of baseline measurements. Additionally, the routine administration of supplemental oxygen to all patients as primary care may have introduced confounding effects on oxygen saturation measurements in the first hours post-WCT. However, significant improvements in oxygen saturation were observed in the first week and first month post-WCT. The MRC Dyspnea Scale and Peak Flow Meter readings demonstrated statistically significant differences between the WCT and control groups at the one-hour, one-week, and one-month follow-up intervals. In contrast, respiratory rate (RR) only differed significantly between the groups in the first hour after intervention, with no significant differences observed in the first week or first month. Correcting tachypnea beside improving dyspnea may have alleviated respiratory muscle fatigue, leading to enhanced breathing depth and strength. Consequently, this may lead to an increase in peak flow rate. These findings suggest a rapid and sustained positive effect of WCT on respiratory function.

Consistent with previous findings by Leroyer et al. (1998) who documented transient declines in respiratory function or heightened bronchial irritability following accidental high-concentration chlorine gas exposure among at-risk workers, the majority of cases showed improvement within three months. However, separate reports suggest the potential for long-term pulmonary complications arising within six months to one year after chlorine exposure (Faria et al., 2021). Notably, our intervention demonstrated a reduction in chlorine-induced RADS recovery time, particularly within the first hour following WCT. This finding suggests that WCT is a potentially valuable early intervention to expedite recovery from RADS.

Furthermore, the absence of symptom reemergence or relapse during the one-month follow-up period strengthens the evidence for sustained improvement in respiratory symptoms following WCT intervention in chlorine-exposed patients. This underscores the importance of prompt intervention and close monitoring during the acute phase of chlorine exposure to optimize patient outcomes and potentially mitigate the risk of future complications.

Despite the incomplete understanding of WCT's mechanism of action, the proposed theoretical frameworks and existing research findings align with the physiopathological mechanisms involved in RADS resulting from chlorine gas exposure (Table 3). Some these potential mechanisms include:

1. Anti-inflammatory effects: The biomolecular mechanism of chlorine gas inhalation toxicity involves inflammation (Jurkuvenaite et al., 2015), triggered by activation of oxidative stress pathways and pro-inflammatory cytokines such asinterleukin 1-beta (IL-1β), tumor necrosis factor alpha (TNF-α), interleukin-8(IL-8), and tumor necrosis factor beta-1 (TNF- β1) (Roux et al., 2005; Samal et al., 2010). Several studies suggest that WCT may exert anti-inflammatory effects by suppressing the production of these pro-inflammatory mediators and oxidative stress factors (Ekrami et al., 2021; El-Domyati et al., 2013; Ke and Long, 2014; Mahdavi et al., 2012; Zhang et al., 2006; Zhang et al., 2018;). This potential mechanism aligns with evidence demonstrating WCT's ability to reduce inflammatory markers like erythrocyte sedimentation rate (ESR), C-reactive protein (CRP), and soluble interleukin-2 receptors (sIL-2R) (Ahmed et al., 2005). Additionally, WCT may possess immunomodulatory properties, potentially influencing inflammatory signaling pathways like NF-κB and enhancing complement system activity, thereby promoting a balanced immune response and potentially bolstering immune defenses (Al-Bedah et al., 2019; Baghdadi et al., 2015; Dons' koi et al., 2016; El-Domyati et al., 2013; Khalil et al., 2013; Soleimani et al., 2020; Widada, 2017). 

2. Enhancement of Alveolar Fluid Clearance (AFC): While direct therapeutic interventions targeting alveolar fluid clearance (AFC) remain elusive (Huppert et al., 2019), growing evidence suggests that WCT may enhance AFC as a potential mechanism for its effectiveness in pulmonary edema. WCT is postulated to improve skin microcirculation (Goodwin and McIvor, 2011), facilitating the clearance of inflammatory mediators and interstitial lung fluid (Akbari et al., 2013; Jin et al., 2011; Tian et al., 2007). Increasing hydrostatic pressure created at the cupping site, based on the principles of communicating vessels, may influence and elevate the gradient between alveolar capillary blood pressure and alveolar endothelial and epithelial permeability, thereby potentially augmenting AFC (El-Sayed et al., 2013; Wei et al., 2013). Subsequently, it may stimulate lymphatic drainage, promoting the removal of excess fluid and debris from the lungs.

3. Downregulation of capillary endothelial and alveolar epithelial permeability: Chlorine-induced lung injury triggers the release of pro-inflammatory cytokines (Fukuda et al., 2001), compromising vascular endothelial integrity by damaging endothelial cadherin (VE-cadherin) – a protein crucial for maintaining the barrier function of small capillary endothelial membranes (Vestweber, 2008). This disrupts endothelial permeability, leading to the accumulation of fluid within the alveoli of the lungs (Elia et al., 2003). While the findings by Widada (2017), regarding to enhancement of spectrin elasticity in red blood cells following WCT may require further investigation, WCT may offer protective effects on the integrity of the alveolar-capillary barrier through mechanisms yet to be elucidated. 

4. Improvement of lung ventilation: Alveolar fluid clearance (AFC) reduction contributes to interstitial pulmonary edema, ultimately leading to respiratory failure via gas exchange dysfunction (Huppert et al., 2019). This dysfunction arises from osmotic pressure disorders triggered by Na/K-ATPase channel dysfunction due to hypoxemia and hypercapnia. Activated oxidants further exacerbate AFC reduction. Hypoxemia and hypercapnia can directly trigger reactive oxygen species (ROS) generation, leading to alveolar damage characterized by endocytosis and cell necrosis (Briva et al., 2007; Dagenais et al., 2004; Vadász et al., 2007; Vivona et al., 2001). Notably, cupping therapy has been shown to improve gas exchange by increasing arterial blood oxygen saturation (SaO2) in smoker men and COVID-19 patients (Aleyeidi et al., 2015; Ali-Ismail et al., 2021; Hekmatpou et al., 2013; Seydi et al., 2023). Furthermore, respiratory discomfort with tachypnea and high tidal volumes elevate airway hydrostatic pressure, ultimately contributing to cell necrosis (Frank et al., 2002). Interestingly, cupping therapy has demonstrated the potential for reducing hyperventilation and improving pulmonary perfusion in hospitalized children with acute pneumonia (Klepikov, 2018).

5. Rehealing of damaged endothelium and epithelial tissue of alveoli (to prevent damage and secondary complications): Disruption of vectorial ion transport and the resulting insufficient osmotic gradient hinder the timely recovery of alveolar epithelial type I and II cells from necrosis, potentially leading to secondary and long-term complications such as obstructive bronchitis (obliterans bronchitis) (Briva et al., 2007; Pugin et al., 1999; Vadász et al., 2007; Vivona et al., 2001). WCT has demonstrated promise in mitigating both the acute and chronic sequelae of chlorine gas-induced lung injury via improving vascular compliance, increasing the blood flow, and enhancing AFC (Aleyeidi et al., 2015; El-Sayed et al., 2013; Jin et al., 2011; Lee et al., 2010; Refaat et al., 2014; Tian et al., 2007; Wei et al., 2013). Table 2 summarizes some aspects of WCT's potential effects relevant to this study.

While further research is necessary to definitively elucidate the mechanisms underlying WCT's efficacy, the proposed anti-inflammatory, AFC-enhancing, and immunomodulatory effects align with the known physiopathology of chlorine-induced RADS. These findings suggest a potential therapeutic role for WCT in RADS, potentially complementing with conventional treatment options. However, it is important to acknowledge that this is a hypothetical explanation based on existing research and theories. Further studies are needed to confirm these proposed mechanisms and establish the clinical efficacy of WCT for chlorine-induced RADS.

Limitations of the present study include the small sample size, attributable to the COVID-19 pandemic, and the unknown concentration of chlorine stimulants due to ethical considerations. Future studies with larger sample sizes and exploration of diverse irritant inhalation agents causing RADS are recommended to elucidate the broader effects of WCT. Additionally, animal studies could be employed to determine minimal chlorine gas concentrations-induced RADSfor future ethically designed human trials.

**Table 1:** Clinical guidelines for Chlorine gas exposure outlined by the Ministry of Health of Iran (2017)

Intervention	Dosage	Indication	Description
Decontamination	Wash face with soap and water; Wash eyes with N/S. 0.9% or clean water for 15-17 minutes	Eye/face contamination	Eye washing should continue until asymptomatic or pH = 8.5
Eye Drops (Tetracaine 0.5%)	1 drop in each eye	Eye contamination or blurred vision	Administer before washing the eyes
Oxygen administration	PaO2 ≥ 60 mm Hg	Respiratory distress symptoms	Limit to 24 hours if FiO2 > 50% due to oxygen toxicity
Nebulized bicarbonate	Sodium bicarbonate (5.7% or 4.8%) 1ml + D.W. 3ml	Early exposure	Use with nebulizer if available
Inhaled bronchodilators	Albuterol: 2 puffs every 25 min; Ipratropium: 2 puffs every 12 hours	Bronchospasm from chlorine exposure	Use with a spacer
Hydration therapy	N/S state (IV)	Nausea with non-oral intake	Limit fluid to prevent ARDS
Antiemetic drugs	Metochlopramide: 10mg/IV/IM; or dimenhydrinate: 50 mg/IV/IM	Nausea/vomiting	Can be repeated (every 4-6 hours) until symptoms resolve
Sedation treatment	Diazepam 5 mg (repeat every 7 min)	Patient agitation	Continue until sedation effect without unconsciousness
Inhaled corticosteroids	Beclomethasone: 2 puffs, repeat after 7 min	Inflammation	
Epinephrine nebulizer	1.5 ml Epinephrine + 4.5 ml D.W.	Decreased SpO2	For severe respiratory distress
N-acetylcysteine (NAC)	IV: 150 mg/kg over 24 hours	Hospitalized cases	

N/S: Normal saline; PaO2: Partial pressure of oxygen; FiO2: Fraction of inspired oxygen; D.W.: Distilled water; min: minutes; IV: Intravenous; ARDS: Acute Respiratory Distress Syndrome.

**Table 2:** Changes in patients' signs in the two groups poisoned with Chlorine gas, before, first hour, first week, and first month post-intervention

**Variables**	**Group**	**Time**				**p-value** ^1^
		**Before intervention**	**first hour after intervention**	**first week after intervention**	**first month after intervention**	
MRC Dyspnea Scale	Control	3.58(±1.73)	3.58(±1.73)	1.92(±0.67)	1.50(±0.52)	0.006*
	Intervention	4.00(±2.41)	2.00(±1.35)	1.25(±0.87)	1.08(±0.29)	0.009*
	p-value^2^	0.632	0.02*	0.046*	0.024*	
Peak flow meter	Control	170.00(±51.70)	165.83(±48.89)	212.50(±37.20)	233.33(±41.19)	0.001>*
	Intervention	201.67(±114.88)	263.33(±88.25)	291.67(±85.90)	325.00(±80.85)	0.001*
	p-value^2^	0.393	0.003*	0.008*	0.002*	
Sp O2	Control	96.67(±2.35)	96.58(±2.39)	95.33(±2.15)	95.33(±2.02)	0.283
	Intervention	97.50(±1.31)	96.83(±2.33)	97.42(±2.02)	98.00(±1.28)	0.179
	p-value^2^	0.295	0.798	0.023*	0.001*	
RR	Control	36.00(±8.49)	34.67(±9.09)	16.33(±1.51)	15.33(±1.63)	0.001*
	Intervention	30.67(±15.47)	20.00(±11.03)	16.00(±9.88)	14.00(±5.06)	0.001*
	p-value^2^	0.485	0.026*	0.065	0.093	
PR	Control	83.50(±11.85)	83.67(±11.68)	74.33(±8.71)	72.75(±5.05)	0.014*
	Intervention	83.75(±19.35)	82.42(±18.57)	77.17(±11.10)	75.83(±7.74)	0.253
	p-value^2^	0.970	0.845	0.494	0.260	
SBP	Control	122.08(±18.27)	122.50(±17.25)	120.00(±13.31)	120.00(±12.79)	0.801
	Intervention	118.75(±17.47)	116.67(±11.74)	117.08(±6.56)	117.92(±8.11)	0.894
	p-value^2^	0.652	0.343	0.503	0.638	
DBP	Control	79.58(±6.20)	79.58(±6.20)	79.17(±7.33)	78.67(±3.75)	0.889
	Intervention	79.17(±8.21)	78.33(±5.77)	79.58(±5.42)	78.33(±5.77)	0.849
	p-value^2^	0.890	0.614	0.876	0.868	

Data are reported as mean±(SD), SD: Standard deviation; Control: Receiving conventional treatment; Intervention: Receiving conventional treatment plus wet cupping (Hijamat); SpO2: Oxygen saturation percentage; RR:Respiratory Rate; PR: Pulse rate;SBP: SystolicBlood pressure; DBP:Diastolic blood pressure; 1: The within-group p-value analyzed using a paired sample t-test in each group (12 patients in each group); 2: The between-group p-value analyzed using an independent sample t-test inter two groups.*p-values that showed significant changes.

**Table 3:** Evidence of the potential therapeutic effects of “Hijamat” (WCT) on improving alveolar fluid clearance (AFC) align the pathogenesis of Chlorine gas poisoning

**Causes of reduction in AFC in Chlorine gas-induced ARDS**	**Ref.**	**Evidence-based results of therapeutic effects of WCT on AFC**	**Ref.**
**Hypoxia/hypercapnia:** hypoxia/hypercapnia can trigger the generation of reactive oxygen species, leading to alveolar damage characterized by endocytosis and cell necrosis.	(Briva et al., 2007; Vadász et al., 2007; Vivona et al., 2001)	1. WCT increased arterial blood oxygen saturation in 110 smokers with COPD symptoms. Sedentary male smoker students improved chest expansion, oxygen saturation, blood pressure, and pulse rate following WCT.	(Aleyeidi et al., 2015; Ali-Ismail et al., 2021; Hekmatpou et al., 2013;)
		2. WCT reduces the concentration of oxidative stress factors by effectively eliminating oxidants from the body.	(Tagil et al., 2014)
		3. WCT is a treatment method that removes blood oxidants or free radicals through the skin's surface.	(Akbari et al., 2013)
**Excessive inflammatory response:** Excessive inflammatory response can lead to the disruption of alveolar barrier function by inflammatory molecules. This disruption increases alveolar-capillary permeability, resulting in the induction of alveolar edema due to the accumulation of fluid.	(Jurkuvenaite et al., 2015)	1. WCT increased the number of blood granulocytes and enhanced the activity of the complement system.	(Khalil et al., 2013)
		2. WCT decreased the number and activity of natural killer cells (NKc), indicating a regulatory effect on inflammation.	(Dons' koi et al., 2016)
		3. WCT can increase T CD8+ and NK cells.	(Widada, 2017)
		4. WCT has been shown to increase the levels of T CD8+ and NK cells. Additionally, it has been observed to elevate the number of NK cells and white blood cell (WBC) count while reducing inflammatory factors such as erythrocyte sedimentation rate (ESR), C-reactive protein (CRP), and soluble interleukin 2 receptors (SIL 2R).	(Ahmed et al., 2005)
		5. WCT has been shown to increase Th2 and T regulatory (Treg) cells while reducing the levels of Th1 and Th17 cells, as well as the Th1/Th2 ratio, indicating an inhibitory effect on inflammation. Furthermore, WCT has been found to elevate the Treg/Th17 ratio, suggesting a positive regulatory effect on the immune system. Additionally, WCT may enhance tolerance to self-antigens and decrease the propensity for the progression of autoimmune diseases.	(Soleimani et al., 2020)
		6. WCT has been observed to reduce the levels of autoantibodies, inflammatory mediators, and serum ferritin.	(Baghdadi et al., 2015)
**High level of** **pro-inflammatory cytokines:** **(**IL-1β, IL-8, TNF-α, TGF- β1) Elevated levels of pro-inflammatory cytokines, including IL-1β, IL-8, TNF-α, and TGF-β1, contribute to the pathogenesis of alveolar injury by reducing levels of ion transport proteins in alveolar edema fluid.	(Dagenais et al., 2004; Elia et al., 2003; Frank et al., 2002; Fukuda et al., 2001; Olman et al., 2004; Pugin et al., 1999; Roux et al., 2005; Samal et al., 2010; Ware and Matthay, 2000;)	1. WCT may influence the immune system by inducing local inflammation, activating the complement system, and increasing the levels of immune products such as interferons.	(Al-Bedah et al., 2019)
		2. WCT decreased serum levels of IgEand IL-2 and increased serum C3 levels.	(El-Domyati et al., 2013)
		3. Compared to the control group (loratadine, budesonide), WCT significantly decreased serum levels of IL-4 and IgE.	(Ke and Long, 2014)
		4. WCT increased serum levels of IFN-γ, IL-2, T.CD4+, IgA, IgM, IgG, and complement proteins while reducing IL-4, IL-10, IgE, and T.CD8+.	(Zhang et al., 2006)
		5. WCT reduced inflammatory markers TNF-α and IL-6 induced by vigorous exercise.	(Ekrami et al., 2021)
		6. There are higher concentrations of IFN-γ and IL-4 in cupping blood serum compared to venous blood.	(Mahdavi et al., 2012)
		7. WCT significantly increased anti-inflammatory lipids while decreasing pro-inflammatory cytokines IL-6 and TNF-αinduced by lipopolysaccharide. It also downregulated IL-6 and TNF-α expression and reduced their production in macrophages.	(Zhang et al., 2018)
**Excess Endothelial and Epithelial Permeabilities** **:** Excessive endothelial and epithelial permeabilities lead to the leakage of inflammatory factors, water, and blood components into the alveolar space caused by the disruption of VE-cadherin bonds in capillary endothelial cells.	(Vestweber, 2008)	WCT increased spectrin elasticity during the 15 days following therapy.	(Widada, 2017)
**Biomechanical stress:** High tidal volumes elevate airway hydrostatic pressures, leading to cell necrosis.	(Frank et al., 2002)	WCT reduced hyperventilation and improved pulmonary perfusion in hospitalized children with acute pneumonia (A Comparative rheopulmonographystudy(.	(Klepikov, 2018)
**Slow Resolution:** Insufficient osmotic gradient caused by the disturbance of vectorial ion transport due to damage of alveolar epithelial type I and type II cells.	(Briva et al., 2007; Vadász et al., 2007; Vivona et al., 2001)	1. An increase in hydrostatic pressure at the cupping site, according to the laws of Communicating vessels, affects and increases the gradient of alveolar capillary blood pressure and alveolar endothelial and epithelial permeability, increaseing alveolar fluid clearance (AFC). This pressure is 16 to 43 times the net glomerular pressure (10 mm Hg outward).	(El-Sayed et al., 2013; Wei et al., 2013)
		2. WCT has shown a significant effect in reducing acute blood pressure by improving vascular compliance.	(Aleyeidi et al., 2015; Lee et al., 2010; Refaat et al., 2014;)
		3. Cupping dilates local capillaries and increases skin blood flow.	(Jin et al., 2011, Tian et al., 2007)

WCT: Wet Cupping Therapy; AFC: Alveolar Fluid Clearance; ARDS: Acute Respiratory Distress Syndrome; VE-cadherin: Vascular Endothelial cadherin
